# Domestic Correspondence

**Published:** 1883-11

**Authors:** F. A. Dunsmoor

**Affiliations:** Dean; Minneapolis, Minn.


### Domestic Correspondence.

MINNEAPOLIS, MINN., October 5, 1883.

A meeting of the faculty of the Minnesota College Hospital was held at the office of the Dean of the college October 4, to pay respect to the memory of Prof. Wm. H. Byford, Jr., who died at his residence on Second Avenue, Thursday morning of this week.

At which it was

*Resolved*, That as it hath pleased a loving and gracious Father to permit Dr. Byford after a long and protracted period of great suffering to lay gently down the burden of life; that in his early death the college to which he was devotedly attached suffers the great loss of an efficient, wise and honorable instructor, whose efforts in its behalf and in the cause of medical education in general, more constant and always marked by that wisdom and culture which is born or a wide experience and a liberal mind.

*Resolved*, That all his intercourse wtth his friends and professional brethren was marked by a high sense of honor and courtesy, and in his early and sudden death we lose a wise counsellor and helpful friend.

*Resolved*, That we deeply sympathize with his bereaved and sorrowing wife, with his honored father, and afflicted family in this their great sorrow.

*Resolved*, That a copy of these resolutions be sent to his bereaved wife, his sorrowing father and family, and that copies be sent for publication to the local papers and prominent medical journals.

F. A. DUNSMOOR, Dean.

